# The Role of Oxidative Stress in Hyperuricemia and Xanthine Oxidoreductase (XOR) Inhibitors

**DOI:** 10.1155/2021/1470380

**Published:** 2021-03-26

**Authors:** Ning Liu, Hu Xu, Qianqian Sun, Xiaojuan Yu, Wentong Chen, Hongquan Wei, Jie Jiang, Youzhi Xu, Wenjie Lu

**Affiliations:** ^1^Basic Medical College, Anhui Medical University, Hefei 230032, China; ^2^College of Pharmacy, Anhui Medical University, Hefei 230032, China

## Abstract

Uric acid is the end product of purine metabolism in humans. Hyperuricemia is a metabolic disease caused by the increased formation or reduced excretion of serum uric acid (SUA). Alterations in SUA homeostasis have been linked to a number of diseases, and hyperuricemia is the major etiologic factor of gout and has been correlated with metabolic syndrome, cardiovascular disease, diabetes, hypertension, and renal disease. Oxidative stress is usually defined as an imbalance between free radicals and antioxidants in our body and is considered to be one of the main causes of cell damage and the development of disease. Studies have demonstrated that hyperuricemia is closely related to the generation of reactive oxygen species (ROS). In the human body, xanthine oxidoreductase (XOR) catalyzes the oxidative hydroxylation of hypoxanthine to xanthine to uric acid, with the accompanying production of ROS. Therefore, XOR is considered a drug target for the treatment of hyperuricemia and gout. In this review, we discuss the mechanisms of uric acid transport and the development of hyperuricemia, emphasizing the role of oxidative stress in the occurrence and development of hyperuricemia. We also summarize recent advances and new discoveries in XOR inhibitors.

## 1. Introduction

Uric acid is a heterocyclic organic compound with the formula C_5_H_4_N_4_O_3_ (7, 9-dihydro-1H-purine-2,6,8(3H)-trione) and has a molecular mass of 168 Da. Uric acid was first isolated from kidney stones in 1776 by the Swedish chemist Carl Wilhelm Scheele [1]. Then, the Ukrainian chemist Ivan Horbaczewski first synthesized uric acid by melting urea with glycine in 1882 [2]. Uric acid is a diprotic acid with pKa1 = 5.4 and pKa2 = 10.3; thus, it predominately exists as monosodium urate (MSU) ion at physiological pH. In general, the water solubility of uric acid and its related metal salts is rather low and temperature dependent. All these salts exhibit greater solubility in hot water than cold water. In humans and the great apes, uric acid is the endpoint of purine metabolism. Alteration of SUA homeostasis depends on the balance between production, the intricate processes of secretion and reabsorption in the kidney tubule, and excretion in the intestine. It is estimated that approximately 30% of uric acid is excreted by the intestine and renal mechanisms of urate excretion account for the other 70% [3]. In the human kidney, three urate transporters, URAT1/SLC22A12, GLUT9/SLC2A9, and ABCG2/BCRP, play vital roles in the regulation of SUA, and the completion of urate reabsorption and secretion may occur through a complex array of mechanisms taking place in the proximal tubule [3, 4]. Studies have shown that overproduction from hepatic metabolism or renal under excretion or extrarenal under excretion, or both can result in higher serum uric acid (SUA), termed hyperuricemia, which is the main predisposing factor for gout [5]. However, in most mammalian species such as rats and mice, uric acid generated from purine metabolism is further degraded into the more soluble compound allantoin by uricase, an enzyme that is mostly found in the liver. In humans, the uricase gene is crippled by two mutations so that the level of SUA in humans is much higher than other mammals [6, 7].

**Figure 1:**
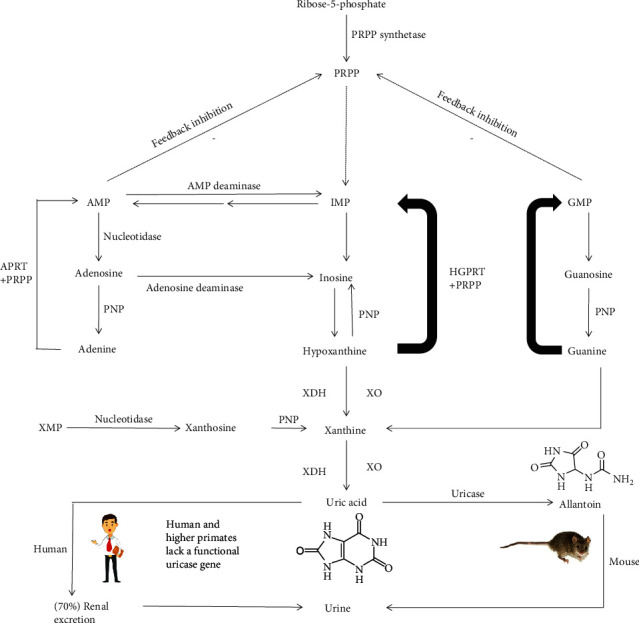
Purine metabolism. Adenosine monophosphate (AMP) is converted to inosine by forming inosine monophosphate (IMP) as an intermediate by AMP deaminase, or by nucleotidase to form adenosine followed by purine nucleoside phosphorylase (PNP) to form adenine; simultaneously, guanine monophosphate (GMP) is converted to guanosine by nucleotidase followed by PNP to form guanine. Moreover, AMP and GMP also have feedback regulation on 5-phosphoribosyl-1-pyrophosphate (PRPP). Hypoxanthine is oxidized to form xanthine by XOR which includes XDH and XO, and the conversion of guanine to xanthine occurs through the action of guanine deaminase. The enzyme hypoxanthine-guanine phosphoribosyl transferase (HGPRT) salvages hypoxanthine to IMP and GMP. In a similar salvage pathway, adenine phosphoribosyl transferase (APRT) converts adenine to AMP. Finally, XOR catalyzes the oxidation of xanthine to uric acid, with the accompanying production of ROS. In most mammalian species such as rats and mice, uric acid generated from purine metabolism is further degraded into allantoin by uricase, an enzyme that is mostly found in the liver. However, in humans and the great apes, uric acid is the endpoint of purine metabolism because the uricase gene is crippled. It is estimated that approximately 30% of uric acid excretion is by the intestine and renal mechanisms of urate excretion account for the other 70%.

One of the most plentiful metabolite classes within a mammalian cell is purines. Purine is a heterocyclic aromatic organic compound that consists of a pyrimidine ring fused to an imidazole ring and is water soluble. Purines are the most widely occurring nitrogen-containing heterocycles in nature and are found in high concentrations in meat and meat products, especially seafood and internal organs. Examples of purine-rich foods include meats, organ meat (such as the liver and kidney), seafood, legumes, yeast, mushrooms, sweetbreads, sardines, brains, mackerel, scallops, and gravy [8, 9]. Higher levels of meat or seafood consumption are associated with an increased risk of gout, whereas proper intake of purine-rich vegetables or protein is not associated with an increased risk of gout [10]. The metabolism of purines is a complex system containing various enzymes. Adenosine monophosphate (AMP) is converted to inosine by forming inosine monophosphate (IMP) as an intermediate by AMP deaminase, or by nucleotidase to form adenosine followed by purine nucleoside phosphorylase (PNP) to form adenine; simultaneously, guanine monophosphate (GMP) is converted to guanosine by nucleotidase followed by PNP to form guanine [4, 7]. Hypoxanthine is then oxidized to form xanthine by XOR (including XDH and XO), and the conversion of guanine to xanthine occurs through the action of guanine deaminase. Finally, XOR catalyzes the oxidation of xanthine to uric acid, with the accompanying production of ROS [11, 12] (Figure 1).

**Figure 2:**
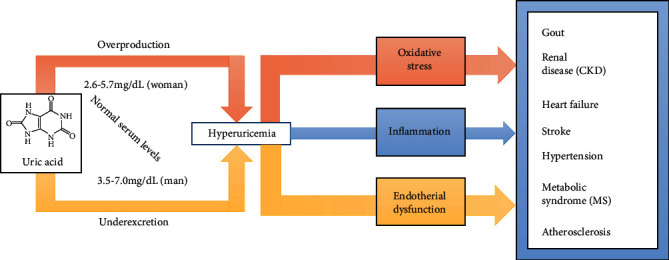
Hyperuricemia and related diseases. Hyperuricemia occurs as a result of increased uric acid production, impaired renal uric acid excretion, or a combination of both mechanisms. In humans, normal SUA levels are 2.6–5.7 mg/dL (155–339 *μ*mol/L) for women and 3.5–7.0 mg/dL (208–416 *μ*mol/L) for men. Moreover, hyperuricemia may cause oxidative stress, inflammation, and endothelial dysfunction, and hyperuricemia is even more of a burden due to its association with multiple comorbidities, including gout, hypertension, cardiovascular disease, chronic kidney disease (CKD), stroke, atherosclerosis, and metabolic syndrome (MS).

Hyperuricemia has become increasingly common over the last few decades, and the burden of hyperuricemia is made heavier by its association with multiple comorbidities, including metabolic syndrome, cardiovascular disease, diabetes, hypertension, and renal disease [13–15]. The association of hyperuricemia with related diseases has been described since the late 19th century. Although the importance of these associations remains controversial, increasing data from prospective studies suggest that hyperuricemia is a key risk factor for developing cardiovascular disease or other diseases. However, we still need more evidence to prove whether lowering uric acid levels would be of clinical benefit in the prevention or treatment of these diseases (Figure 2).

Oxidative stress can be defined as the condition in which excessive production of reactive oxygen species (ROS) occurs. In many disease states, oxidant-producing enzymes, the major sources of ROS, are upregulated [16]. Recent studies have demonstrated that asymptomatic young patients with primary hyperuricemia had significantly higher oxidative stress than healthy persons [17]. In the human body, xanthine oxidoreductases (XORs) are critical enzymes for uric acid production, which includes xanthine oxidase (XO) and xanthine dehydrogenase (XDH). Therefore, XOR has become an effective target of drugs for the treatment of hyperuricemia. At present, multiple XOR inhibitor drugs have been widely used, and more new drugs are being developed such as topiroxostat [18].

This review is aimed at elaborating the pathogenesis of hyperuricemia and summarizing the role of oxidative stress in hyperuricemia-related diseases. Simultaneously, this article reviews the updated information available on the role of XOR inhibition.

## 2. Pathogenesis of Hyperuricemia Focused on Oxidative Stress

### 2.1. Asymptomatic Hyperuricemia

Hyperuricemia (HUA) in adults is defined as a serum uric acid level>420 *μ*mol/L (7 mg/dL) in men and >357 *μ*mol/L (6 mg/dL) in women [19, 20]. On the one hand, a diet rich in purine and/or fructose can lead to an increase in serum uric acid. Briefly, fructose is phosphorylated into fructose 1-phosphate in a reaction catalyzed by fructokinase primarily during fructose metabolism and this reaction decreases the levels of intracellular phosphate and ATP [13]. Next, the enzyme fructose-1-p aldolase breaks fructose 1-phosphate into dihydroxyacetone phosphate (DHAP) and D-glyceraldehyde. When there is a high intake of fructose, phosphorylation into fructose 1-phosphate is fast, but the reaction with aldolase is slow. Thus, fructose 1-phosphate accumulates, and decreased intracellular phosphate level stimulates AMP deaminase (AMPD), which catalyze the degradation of AMP to inosine monophosphate [21]. And then, the purine degradation produces UA [22]. Physiologically, fructose also stimulates UA synthesis from amino acid precursors such as glycine [23]. Moreover, long-term fructose stimulation reduces renal excretion of UA, resulting in elevated serum UA levels [24]. The intake of alcohol and excessive exercise can also cause an increase in the level of serum uric acid. Some malignant tumors also increase the level of serum uric acid after chemotherapeutic drugs are used. On the other hand, more than 90% of hyperuricemia is caused by decreased uric acid excretion [25, 26]. It is characterized by high uric acid levels in the blood, causing deposition of urate crystals in the joints and kidneys. In normal humans, uric acid is excreted in urine. However, uric acid excretion may be impaired by kidney disease, leading to hyperuricemia.

Asymptomatic hyperuricemia is a condition in which the serum urate concentration is elevated (>7 mg/dL in men or >6 mg/dL in women) but there are no symptoms or signs of urate crystal deposition [27]. The increasing prevalence of asymptomatic hyperuricemia may be ascribable to the expanding obesity epidemic, dietary changes, an aging population, and the increasing use of diuretics. In some patients with asymptomatic hyperuricemia, steady hyperuricemia is suggested to be sufficient to trigger MSU crystal deposition and MSU crystals can trigger inflammatory pathways (IL-6 and IL-8) [28, 29]. Numerous epidemiological data have shown the association of asymptomatic hyperuricemia with comorbidities and cardiovascular risk factors; however, proof of a causal link is lacking. Thus, large randomized placebo-controlled trials are still needed to assess the significance of treating asymptomatic hyperuricemia [30–32].

A critical point of view should be realized in that the serum uric acid level is not “the lower the better” when we treat hyperuricemia, and the possible danger of overtreatment of hyperuricemia is an abnormally low level of SUA, termed hypouricemia [33]. Hypouricemia is defined as a serum urate concentration of less than or equal to 2.0 mg/dL [33, 34]. Hypouricemia may occur as a consequence of decreased formation of urate or increased renal clearance of urate. This may be due to a decrease in XOR activity or deficiency of the enzyme pathologically, or the presence of XOR inhibitors, such as allopurinol [35, 36]. A moderate or severe hypouricemia leads to an increase in lipid peroxidation through loss of antioxidant capacity of plasma [37]. Congenital hypouricemia patients may be more prone to develop renal failure, and their condition may result from a number of defects in urate transporters [38]. In a majority of patients, the defects are caused by loss-of-function mutations in the SLC22A12 gene that codes for the urate transporter URAT1 (RHUC1). Furthermore, another key player glucose transporter 9 (GLUT9; RHUC2) in UA homeostasis proved to be central to urate reabsorption. This genetic mutation will lead to renal hypouricemia type 2, a monogenic disease characterized by very low SUA, and high fractional excretion of urate [39, 40]. Identifying these mutations is critical and renal hypouricemia can be asymptomatic until the patients are subjected to strenuous exercise, which can lead to acute renal injury [41]. Research indicates that this occurs due to oxidative damage caused by increased ROS production during exercise leading to renal vasoconstriction and ischemia [42]. Therefore, a significant increase in markers for fibrosis, inflammation, and oxidative stress was seen in hypouricemic mice such as transforming growth factor *β* (TGF-*β*) [43]. Even though hypouricemia is usually a rare and asymptomatic disease in humans, animal and cell research evidence points to a potential mechanism of hypouricemia leading to kidney diseases through inflammatory signaling pathways [41].

### 2.2. The Dual Role of Uric Acid

Several experimental and clinical studies support a role for uric acid as a contributory causal factor in multiple conditions including oxidation and antioxidant effects. It has been shown that in physiological concentrations, UA is a potent antioxidant that may protect endothelial cells from extracellularly generated ROS [44]. In the hydrophilic environment, it scavenges carbon-centered radicals and peroxyl radicals such as peroxynitrite (ONOO^–^); meanwhile, UA is responsible for approximately 50% of serum antioxidant activity and contributes to about 70% of all free radical scavenging activities in human plasma [45]. For example, UA can protect the erythrocyte membrane against lipid peroxidation and lysis induced by t-butyl hydroperoxide [46]. Moreover, UA can react with ONOO^–^ to form uric acid nitration/nitrosation derivatives that can release NO and increase NO bioavailability [47]. UA also chelates transition metals to reduce ion-mediated ascorbic acid oxidation [48]. In the latest study, UA can exert beneficial functions due to its antioxidant properties, which may be particularly relevant in the context of neurodegenerative diseases [49]. UA efficiently scavenges carbon-centered and peroxyl radicals only in hydrophilic conditions to inhibit lipid peroxidation, which is probably a major limitation of its antioxidant function [50, 51].

However, in vivo and cellular studies have demonstrated that depending on its chemical microenvironment, UA cannot scavenge all free radicals, such as superoxide, and becomes a strong prooxidant under hydrophobic conditions [50]. For example, UA-induced aging and death of human endothelial cells are mediated by local activation of oxidative stress [52]. UA forms radicals in reactions with other oxidants, and these radicals seem to target predominantly lipids, low-density lipoprotein cholesterol (LDL), and membranes [51, 53]. UA showed to have antioxidant effects in the presence of native LDL but it induces prooxidant effects after the oxidation of LDL [54]. UA can accelerate the copper-induced peroxidation of human LDL in the presence of preformed lipid hydroperoxides [55]. On the other hand, as mentioned before, UA itself can increase oxidative stress via reduced NOX activation and UA transporter 1 is involved in the generation of this oxidative stress. Furthermore, UA locally activates the RAS, thus producing angiotensin II and subsequently increasing intracellular oxidative stress [56]. Thus, the exact role of UA in oxidative stress is not fully elucidated and may depend on physiological and/or pathological conditions. Overall, hyperuricemia contributes to the progression of many diseases through the oxidant property of UA.

### 2.3. Oxidative Stress and Endothelial Dysfunction in Hyperuricemia

Oxygen radicals are ubiquitous in our body and are generated by normal physiological processes. Oxygen radicals include hydrogen peroxide molecules, hydroxyl radicals, peroxide hydroxyl radicals, alkoxy radicals, and superoxide anion radicals, which are collectively referred to as ROS. Oxidative stress is usually defined as the imbalance between oxidants and antioxidants, with excessive ROS. The pathophysiological effects of ROS depend on the type, concentration, and specific site of production. They are involved in multiple mechanisms including the bactericidal activity of phagocytes, signal transduction, regulation of the cell cycle, inflammatory responses, and the redox state [57, 58]. The latest research results also show that the impaired physiological capacity to thermoregulate with advancing age does increase the risk of oxidative stress under challenging conditions. When energy is limited, the risk to encounter oxidative stress is increasing via a compensation to defend normothermic body temperatures [59]. And then, when the local levels of ROS are high, they cause considerable cellular damage and generate other more reactive radicals. At low concentrations, however, local targeted production of ROS serves as a second messenger system that transmits biological information [16, 60].

Important data regarding the involvement of UA in oxidative stress come from experimental studies. It has been proved that hyperuricemia stimulates nicotinamide adenine dinucleotide phosphate (NADPH) oxidase activity and, subsequently, oxygen species synthesis [61, 62]. Moreover, the major cardiovascular sources of ROS include the enzymes XOR, NADPH oxidase (NOX), and NOS, as well as mitochondrial cytochromes and hemoglobin. As regards the production of ROS by XO, under conditions of oxidative stress, XO activity prevails to XDH activity resulting in further ROS production. In cultures of endothelial cells, NOX maintains XO levels and XO is responsible for increased ROS production [63]. Also, ROS-“activated” XO remains as the main source of ROS for the development of diabetic nephropathy and experimental studies have shown that inhibition of XO ameliorates diabetic nephropathy [64]. These results indicated that the interconnection between urate metabolism and ROS production in hypertension and diabetes mellitus also raise the possibility that XO inhibitors may reduce oxidative stress independently of serum UA levels. However, related findings also suggest that hyperuricemia in the Zucker diabetic fatty (ZDF) rat model of obesity and the metabolic syndrome is not caused by renal oxidative stress [65]. On the other hand, UA has been found to stimulate increases in NOX-derived ROS production in various cells, such as adipocytes and vascular endothelial cells [66, 67]. Some results also demonstrated that UA stimulates proliferation, angiotensin II production, and oxidative stress in vascular smooth muscle cells (VSMCs) through the tissue renin-angiotensin system (RAS) [66]. According to previous research, aldose reductase (AR) plays a vital role in the oxidative stress-related complications of diabetes [68]. And Zhang et al. found a significant relationship between hyperuricemia-induced endothelial dysfunction and AR-mediated oxidative stress in human umbilical vein endothelial cells (HUVECs) [69]. Hyperuricemia induced endothelial dysfunction via regulation of AR, while inhibition of AR could restore endothelial function [70]. Meanwhile, mitochondria are the center of intracellular energy metabolism and the main site of oxidative phosphorylation, in which ROS are generated by electron transfer from the electron transport chain complex to O_2_ [71]. It has been reported that renal oxidative stress induced by hyperuricemia promoted mitochondrial functional disturbances and decreased ATP content in rats, which represent an additional pathogenic mechanism induced by chronic hyperuricemia [72]. In addition, uric acid-induced endothelial dysfunction is associated with mitochondrial alterations and decreased intracellular ATP production [73].

In related studies of intracellular mechanisms, endothelial cells secrete various vasoactive substances to regulate the relaxation and contraction of blood vessels, including the potent vasoconstrictor endothelin 1 (ET-1) and the effective vasodilator nitric oxide (NO) [74]. NO has become a basic signaling device and a potent mediator of cellular damage in a wide range of conditions [44, 75]. Accumulating evidence indicates that UA impacts endothelial function through a decline in NO release and endothelial nitric oxide synthase (eNOS) activity, which subsequently decreases NO bioavailability [76–79]. L-arginine is the substrate of eNOS and is converted to NO in mammalian endothelial cells. Research showed that UA could enhance the affinity of L-arginine to arginase, an enzyme degrading L-arginine, which reduced the availability of the substrate for NO synthesis [80]. RAS activation by increased UA may also impair endothelial NO production [81]. The decrease in NO bioavailability promotes endothelial dysfunction increases vascular tone and may contribute to arterial stiffness [66].

**Figure 3:**
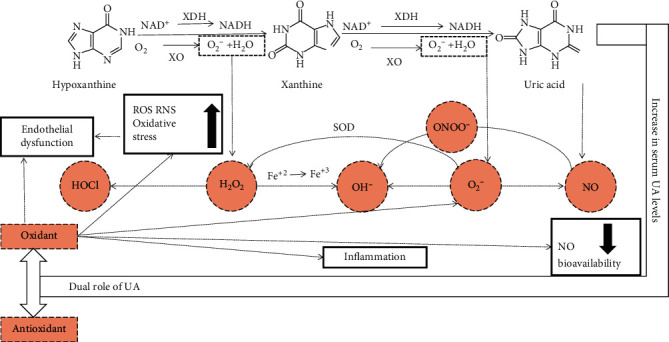
Uric acid and oxidative stress. XOR, which is a critical enzyme in the production of uric acid, can produce O_2_^–^ and H_2_O_2_. Then, the reaction between O_2_^–^ and NO reduces NO bioavailability, which is a main cause of endothelial dysfunction. Moreover, O_2_^–^ can undergo the disproportionation reaction into H_2_O_2_ by superoxide dismutase (SOD), and O_2_^–^ and H_2_O_2_ can also be converted to the more cytotoxic oxidants peroxynitrate (ONOO^–^), hydroxyl anion (OH^–^), and hypochlorous acid (HOCl), which are more harmful to cells. These high levels of ROS result in oxidative stress. On the other hand, several experimental and clinical studies support a role for uric acid as a contributory causal factor in multiple conditions, including oxidation and antioxidant effects. The critical point is that UA becomes a strong prooxidant in the intracellular environment and is associated with various factors, such as inflammation and endothelial dysfunction.

XOR, which is a critical enzyme in the production of uric acid, can produce O_2_^–^ and H_2_O_2_. O_2_^–^ is an oxidative compound that damages the extracellular matrix, increasing the permeability of the microvasculature [82]. Then, the reaction between O_2_^–^ and NO reduces NO bioavailability. In fact, the reaction between O_2_^–^ and NO is faster than O_2_^–^ dismutation by superoxide dismutase (SOD). Moreover, O_2_^–^ and H_2_O_2_ can also be converted to the more cytotoxic oxidants peroxynitrate (ONOO^–^), hydroxyl anion (OH^–^), and hypochlorous acid (HOCl), which are more harmful to cells (Figure 3) [83]. In the kidney, superoxide can also be produced by XDH or NOX [84]. Finally, these ROS produce oxidative stress, which damages proteins, lipids, DNA, and RNA and participates in a wide range of cellular processes including cellular signaling, cardiovascular disease (CVD), inflammation, aging, and cancer [85].

Some natural compounds that can treat oxidative stress induced by hyperuricemia have also been discovered in previous studies. It has been reported that iptakalim, an ATP-sensitive potassium channel opener, could improve endothelial dysfunction and defend against hyperuricemia [86]. And using stevia (Stevia rebaudiana Bertoni) byproduct, named stevia residue extract (STVRE), to treat hyperuricemia, Arshad Mehmood et al. confirmed in a recent study that the STVRE remarkably attenuated oxidative stress mediated by UA and downregulated inflammatory-related response markers such as COX-2, NF-*κ*B, PGE2, IL-1*β*, and TNF-*α* [87]. In addition, related research has shown that UA-induced oxidative stress may activate the Notch 1 pathway, which is involved in the UA inflammatory process. And (-)epigallocatechin-3-gallate (EGCG), a flavanol derived from green tea extracts with antioxidant effects, can prevent the UA-induced inflammatory effect of human umbilical vein endothelial cells (HUVEC) [88].

## 3. Xanthine Oxidase Inhibition Studies

**Figure 4:**
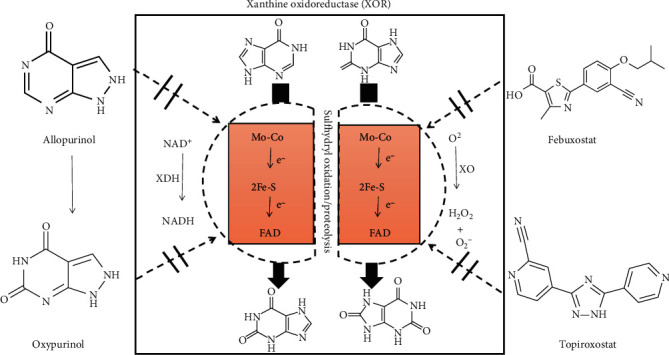
Chemical structure of xanthine oxidoreductase (XOR) and XOR inhibitors. Xanthine oxidase (XOR) is the enzyme that catalyzes the oxidation of hypoxanthine to xanthine and xanthine to uric acid. XOR contains two forms: xanthine dehydrogenase (XDH) and xanthine oxidase (XO). XDH prefers NAD^+^ as the substrate, and XO prefers O_2_. XOR has 2 flavin molecules (FAD), 2 molybdenum atoms, and 8 iron atoms bound per enzymatic unit. The molybdenum atoms are the active sites of the enzyme, and the iron atoms are part of the [2Fe-2S] ferredoxin iron-sulfur clusters and participate in electron transfer reactions. XOR is a critical target of drug action in the treatment of hyperuricemia. XOR inhibitors are potentially effective drugs to control the related diseases and dysfunctions and include allopurinol, oxypurinol, febuxostat, and topiroxostat.

XOR is the rate-limiting enzyme in purine catabolism and is widely distributed among species [89]. XOR contains two forms: XDH and XO. Most of the protein in the liver exists in a form with XDH activity, but it can be converted to XO by reversible sulfhydryl oxidation or by irreversible proteolytic modification. XOR catalyzes the last 2 steps of purine catabolism including the oxidation of hypoxanthine to xanthine and the oxidation of xanthine to uric acid, with the accompanying production of ROS [90–94]. XDH prefers nicotinamide adenine dinucleotide (NAD^+^) as the substrate and XO prefers O_2_. In the process of uric acid production, NAD^+^ accepts XDH transfer electrons to form hydrogen nicotinamide adenine dinucleotide (NADH). XO uses molecular oxygen as an electron acceptor to replace NAD^+^, resulting in the formation of the oxygen free radical superoxide anion (O_2_^−^) and other ROS, further causing oxidative stress [95] (Figure 4).

XO is a versatile molybdoflavoprotein that is widely distributed, occurring in milk, the heart, the liver, the kidney, the vascular endothelium, and insects [96]. The protein is a large molecule with a molecular weight of 270 kDa and has 2 flavin molecules (FAD), 2 molybdenum atoms, and 8 iron atoms bound per enzymatic unit [94]. The iron atoms are part of the [2Fe-2S] ferredoxin iron-sulfur clusters and participate in electron transfer reactions [97]. In addition to the ruthenium derivative as an electron donor, pteridine derivatives and aldehydes (formation carboxylic acid) can be used as electron donors. The active site of XO is composed of a molybdopterin unit with the molybdenum atom, which is coordinated by terminal oxygen, sulfur atoms, and a terminal hydroxide. In the reaction with xanthine to form uric acid, an oxygen atom is transferred from molybdenum to xanthine, and peroxide is formed [98], whereby several intermediates are assumed to be involved. XDH belongs to the group of molybdenum-containing hydroxylases involved in the oxidative metabolism of purines and the enzyme is a homodimer. Related research demonstrates that hepatocyte XDH expression is a critical factor of systemic UA homeostasis and plasma XOR activity [99]. The difference between XO and XDH is that oxidase only reduces oxygen, but dehydrogenase can not only reduce oxygen but also reduce NAD^+^ and binds more closely with NAD^+^. However, both forms of enzymes catalyze the reaction of hypoxanthine to xanthine and xanthine to uric acid [11].

XOR could contribute to the pathogenesis of metabolic syndrome through oxidative stress and the inflammatory response induced by XOR-derived ROS and UA [89, 100]. Moreover, the serum level of XOR is associated with TG/HDL-C ratio, fasting glycemia, fasting insulinemia, and the insulin resistance index. In addition, XOR is implicated in preadipocyte differentiation and adipogenesis. On the other hand, the cytocidal action of XOR products has been claimed in relation to tissue damage, especially damage induced by hypoxia and ischemia [90]. Furthermore, XOR and UA have also been implicated in the progression of hypertension and oncogenesis because XOR is able to catalyze the metabolic activation of carcinogenic substances [91, 101]. However, XOR activity creates both oxidant and antioxidant products; in some circumstances, they may have antioxidant protective outcomes. In particular, uric acid may have a protective as well as a detrimental role in vascular alterations, thus justifying the multidirectional effects of XOR inhibition [100].

In summary, XOR, the enzyme that catalyzes the terminal steps in urate production, is a critical target of drug action in the treatment of hyperuricemia. XOR inhibitors are potentially effective drugs to control these related diseases and dysfunctions. Here, we will introduce some classic XOR inhibitors as well as novel inhibitors and related applications.

### 3.1. Allopurinol and Oxypurinol

Allopurinol (4-hydroxypyrazolo (3,4-d) pyrimidine) was the first XOR inhibitor drug approved by the US Food and Drug Administration (FDA) in 1966 for the treatment of gout and primary and secondary hyperuricemia [102]. Allopurinol, a purine analog, is widely used in the management of multiple disorders including gout, kidney stones, inflammatory bowel disease, and certain enzyme (hypoxanthine-guanine phosphoribosyltransferase) disorders that lead to the overproduction of urate, such as Lesch–Nyhan syndrome [103, 104]. In terms of mechanism, inhibition of xanthine oxidase also causes an increase in hypoxanthine and xanthine in addition to a reduction in uric acid formation. Then, some purine ribotide levels, such as adenosine and guanosine monophosphate levels, are increased, which may cause negative feedback of amidophosphoribosyl transferase, the first and rate-limiting enzyme of purine biosynthesis.

Allopurinol is hydrolyzed by XO to produce oxypurinol, which is the active metabolite of allopurinol and an inhibitor of XO. Oxypurinol inhibits XOR by binding to molybdenum in the enzyme [105]. Allopurinol is almost completely metabolized to oxipurinol within two hours of oral administration, whereas oxipurinol is slowly excreted by the kidneys over 18–30 hours [106]. In addition, aldehyde oxidase (AO) is also an important enzyme in the metabolism of allopurinol and contains molybdenum in its protein structure like XOR. It can also catalyze the oxidation of both cytochrome P450 (CYP450) and monoamine oxidase (MAO) intermediate products [107, 108].

Although allopurinol has been used widely for many years, allopurinol is still subject to continued investigation in the pursuit of better effective health outcomes for patients with gout or hyperuricemia. Allopurinol can be an effective urate-lowering therapy when adequate doses are used [109]. The use of allopurinol, however, can cause adverse effects, ranging from a mild form of allopurinol hypersensitivity to severe adverse reactions involving a rash combined with eosinophilia, leukocytosis, fever, hepatitis, and progressive kidney failure. Serious adverse reactions associated with allopurinol are feared owing to the high mortality [109]. Allopurinol hypersensitivity syndrome (AHS), a feared complication of allopurinol, has been discovered to be at great risk and the mortality rate of AHS is approximately 14% [103, 110]. Meanwhile, its safety in pregnancy has been debated due to reports on possible teratogenicity [111]. In addition, allopurinol may cause some side effects, such as renal stones and neurological disorders, due to xanthine and hypoxanthine accumulation [112].

Allopurinol can not only treat hyperuricemia but also has a significant effect on the treatment of other diseases. Recent studies suggest that cardiovascular disease and mortality, chronic kidney disease, prostate cancer, and manic symptoms are reduced in patients with gout treated with allopurinol [113–116]. Furthermore, allopurinol has analgesic and anti-inflammatory properties [117]. Moreover, in the latest study, allopurinol reduced oxidative stress and activated nuclear factor erythroid 2-related factor 2 (Nrf2)/p62 to attenuate diabetic cardiomyopathy in rats [118].

### 3.2. Febuxostat: A Nonpurine Xanthine Oxidase Inhibitor

In recent years, the nonpurine inhibitor febuxostat was approved in Europe and the USA for the treatment of hyperuricemia and gout. Febuxostat (2-(3-cyano-4-isobutoxy-phenyl)-4-methyl-1,3-thiazole-5 carboxylic acid) is a nonpurine XOR-inhibitor drug with a chemical structure that is different from allopurinol. It is a weak acid (pKa 3.3), whose unionized form is highly lipid soluble and is expected to have good oral availability (84% bioavailability) [119]. In contrast to oxipurinol, febuxostat does not form a covalent bond with molybdenum [120]. Febuxostat is clearly an effective uric acid-lowering agent, and the maximal effect of febuxostat has been shown to be below 300 mmol/L and has durable maintenance for long-term treatment [121]. In addition, febuxostat is associated with ROS production and antioxidant capacity [122, 123]. The accompanying hypouricemic effects were increases in serum xanthine concentrations and urinary hypoxanthine excretion and decreases in urinary uric acid excretion, confirming inhibition of xanthine oxidase activity by febuxostat. Meanwhile, dose individualization is required to achieve target plasma urate values [124, 125]. Furthermore, in clinical trials, it was recently approved at daily doses of 80 to 120 mg/day in comparison with the dosage of allopurinol (300 mg/day) due to its selectivity towards xanthine oxidase. In a recent study of Japanese patients, verinurad, a high-affinity inhibitor of the URAT1 transporter, combined with febuxostat resulted in greater and more consistent SUA lowering in subjects with gout or hyperuricemia [126].

The most commonly reported adverse drug reactions are liver function abnormalities, diarrhea, arthralgia, headache, and nausea. Simultaneously, not the same as allopurinol, allergy and hypersensitivity reactions rarely occur in patients receiving febuxostat. Hepatic conjugation and oxidative metabolism were the major pathways of elimination of febuxostat. Its pharmacokinetics and pharmacodynamics are not significantly altered in patients with moderate hepatic impairment [127, 128]. The urate-lowering effect of febuxostat is unaltered in patients with renal impairment, so the dose of this drug does not need to be adjusted in patients with mild to moderate renal impairment [129]. In summary, febuxostat is a safe and potent hypouricemic agent in humans, and its adverse events are mild and self-limited.

### 3.3. Topiroxostat

Febuxostat was followed by another potent XO inhibitor called topiroxostat (4-[5-(4-pyridinyl)-1H-1,2,4-triazol-3-yl]-2-pyridinecarbonitrile), which serves as a suicide substrate of XOR [130]. Topiroxostat was approved by the Pharmaceutical and Food Safety Bureau in Japan for the treatment of hyperuricemia and gout in 2013 [131]. It also has similar structural features as febuxostat. Topiroxostat transfers two electrons to XOR, forming a stable complex with molybdenum. In the crystal structure of the complex of XO and topiroxostat, topiroxostat forms a covalent bond with molybdenum and generates various interactions through the effect of oxygen, including hydrogen bonding with surrounding amino acids, hydrophobic interactions, and *π*–*π* stacking interactions [132]. A dose-dependent serum urate-lowering efficacy of topiroxostat was observed in Japanese hyperuricemic male patients with or without gout [133]. Moreover, topiroxostat effectively reduced the serum urate level in hyperuricemic patients with stage 3 CKD in a recent study [134]. Furthermore, topiroxostat is postulated to exert a renoprotective effect. The renoprotective effects of topiroxostat could be attributed to inhibition of XO and suppression of intracellular UA production. For example, related results have shown that topiroxostat ameliorates kidney injury in puromycin aminonucleoside nephrosis rats by reducing oxidative stress and the UA concentration [135, 136]. However, febuxostat had stronger renoprotective and antioxidant effects than topiroxostat in patients with hyperuricemia and chronic kidney disease (CKD) [137].

### 3.4. Novel Xanthine Oxidase Inhibitors

In the last decade, in addition to the approved XOR inhibitor drugs including allopurinol, febuxostat, and topiroxostat, there has been a continuous effort to develop new XOR inhibitor drugs. The reasons are mainly about twofold. On the one hand, hyperuricemia has been found to be associated with various conditions such as cardiovascular disease and renal diseases. On the other hand, current drugs are associated with certain adverse effects. In recent years, many novel structures of drugs have emerged [105, 138]. Describing the chemical diversity of XOR inhibitors, we classified them into two main groups: purine-like inhibitors and nonpurine inhibitors. In terms of purine-like inhibitors, a common method is to make small changes to the structure of the natural substrate of an enzyme to obtain structurally similar analogs. The introduction of new substituents to a natural substrate produces a better affinity towards the enzyme. Based on xanthine, new purine-like analogues were reported in some related studies such as the newly synthesized 8-(n-hexylthio) xanthine and the xanthine derivative 1,3-dipropylxanthine substituted benzenesulfonic acid, both of which showed better potency than allopurinol [139, 140]. One of the best irreversible inhibitors of hypoxanthine derivatives was 8-(m-(p-fluorosulfonylbenzamido)benzylthio) hypoxanthine, which inhibited 50% of the related enzyme [141]. 2-Alkylhypoxanthines are also hypoxanthine analogs [140]. There are also inhibitors based on other chemical structures. In 1999, 6-formylpterin was demonstrated to be a valid inhibitor belonging to the pteridine analogs [142]. In recent years, purine-like analogs have been synthesized, such as N-(1,3-diaryl-3-oxopropyl) amides [143], 5,6-dihydropyrazolo/pyrazolo[1,5-c] quinazo line derivatives [144], and a novel potent xanthine oxidase inhibitor, 3-nitrobenzoyl 9-deazaguanine (LSPN451) [145]. However, the abovementioned limitations associated with allo/oxypurinol and some potentially fatal adverse effects led to the search for nonpurine XO inhibitors [132].

The structure of 2-aryl-1-arylmethyl-1H-benzimidazoles was investigated by Nile et al. in 2013, and all analogs exhibited activity comparable to allopurinol [146]. In 2014, a series of naphthopyrans catalyzed by silica supported fluoroboric acid was synthesized by Sharma et al. as a new nonpurine XO inhibitor [147]. Then, in 2015, imidazole derivatives similar in structure to febuxostat were synthesized by Chen et al. and included 2-(3-cyano-4-isobutyloxyphenyl)-1-hydroxy-4-methyl-1H-imidazole-5-carboxylic acid and 2-(3-cyano-4-isobutyloxyphenyl)-1-methoxy-4-methyl-1H-imidazole-5-carboxylic acid [148]. In the same year, Song et al. discovered a series of novel 2-(indol-2-yl)thiazole derivatives as XO inhibitors. Moreover, among the compounds, 2-(7-nitro-5-isopropoxy-indol-2-yl)-4-methylthiazole-5-carboxylic acid exhibited potent XO inhibitory activity (IC_50_ value: 5.1 nM) and excellent uric acid-lowering activity in a hyperuricemic rat model [149]. Among numerous inhibitors, Y-700 (1-(3-cyano-4-neopentyloxyphenyl)-1H-pyrazole-4-carboxylic acid) was identified as the compound with the best IC_50_ (5.8 nM compared to 260 nM for allopurinol) and exhibited inhibitory activity of a mixed type. Similar to febuxostat, Y-700 exhibited more potent and longer-lasting hypouricemic activity than allo/oxypurinol [139, 150]. Moreover, related results suggest that Y-700 is a useful agent for the prevention of colon tumorigenesis [151].

Although febuxostat has fewer side effects, febuxostat and allopurinol still have some adverse reactions such as skin rashes, hepatitis, nephropathy, fatal liver necrosis, and allergic reactions. Hence, alternative medicines with fewer side effects are required to tackle UA disorders. Plants have been used as a medicinal source, and natural medicines have the potential to perform beneficial functions with fewer side effects than synthetic drugs; thus, researchers have focused on natural derivatives for the development of novel XOR inhibitors [152, 153]. Flavones, coumarins, and curcumin represent the class of secondary metabolites possessing xanthine oxidase inhibitory potential [154, 155]. Quercetin, one of the most abundant flavonoids in the daily diet, is a natural flavonol that possesses strong XOR inhibitory activity [156]. In another study, Ding et al. discussed that hydroxycinnamic acids are the phenolic compounds in many plants and exhibited weak XOR inhibitory activity [157]. In addition, several tannins may also inhibit the activity of XOR [158]. Recently, related study found that 3,4-dihydroxy-5-nitrobenzaldehyde (DHNB), a derivative of the natural substance protocatechuic aldehyde, potently inhibited XO activity, which was similar to that of allopurinol [159]. Therefore, a plethora of bioactive compounds in plants inhibit the XOR enzyme close to the levels of allopurinol inhibition such as luteolin, quercetin, isorhamnetin, galangin, chrysin, prosapogenin, and cajaninstilbene acid.

In summary, the understanding of the cellular and molecular mechanisms of XOR inhibitors has increased dramatically and these inhibitors may have played a vital role in hyperuricemia and related diseases.

## 4. Conclusions

In recent years, the prevalence of hyperuricemia has increased worldwide. More studies have demonstrated that hyperuricemia is associated with multiple diseases, including gout, cardiovascular disease, and renal disease. Uric acid, as the metabolic end product of purine metabolism in humans, is closely related to the generation of ROS, which play a vital role in these pathophysiological processes. XOR is the rate-limiting enzyme in purine catabolism that catalyzes the oxidation of hypoxanthine to xanthine and xanthine to uric acid with ROS production. XOR is a critical target of drug action in the treatment of hyperuricemia. Thus, researchers in various countries have developed a number of inhibitors that inhibit the activity of XOR, allopurinol, febuxostat, topiroxostat, and many natural compounds with fewer side effects. However, there is a long way to go in clarifying the mechanisms of ROS production and the role of hyperuricemia in other than gout diseases and developing better drugs to treat hyperuricemia.

In our discussion, oxidative stress has a profound impact on the development of hyperuricemia from a certain level. This is an entry point for clinical research and drug development, including related research on hyperuricemia and mitochondria, lipid metabolism, and inflammation. Technologies such as metabolomics, lipidomics, and single-cell transcriptomics enable us to further study the occurrence and development of its mechanism. We can understand how high uric acid affects the oxidation, metabolic disorders, and apoptosis of different cells through these cutting edge technologies. They will help us to accurately treat hyperuricemia and related diseases. At the same time, xanthine oxidase inhibitors are also worthy of more research. Related studies have reported that febuxostat exerts an anti-inflammatory action and protects against diabetic nephropathy development in KK-Ay obese diabetic mice [160]. This is undoubtedly a major breakthrough for patients with hyperuricemia and diabetes. Thus, a conventional drug in new use will also be a key issue in experimental research.

## References

[1] Scheele C. (1776). Examen chemicum calculi urinarii. *Opuscula*.

[2] Horbaczewski J. (1882). Synthese der Harnsäure. *Monatshefte für Chemie*.

[3] So A., Thorens B. (2010). Uric acid transport and disease. *The Journal of Clinical Investigation*.

[4] Maiuolo J., Oppedisano F., Gratteri S., Muscoli C., Mollace V. (2016). Regulation of uric acid metabolism and excretion. *International Journal of Cardiology*.

[5] Dalbeth N., Merriman T. R., Stamp L. K. (2016). Gout. *The Lancet*.

[6] Wu X. W., Lee C. C., Muzny D. M., Caskey C. T. (1989). Urate oxidase: primary structure and evolutionary implications. *Proceedings of the National Academy of Sciences of the United States of America*.

[7] Mandal A. K., Mount D. B. (2015). The molecular physiology of uric acid homeostasis. *Annual Review of Physiology*.

[8] Kaneko K., Aoyagi Y., Fukuuchi T., Inazawa K., Yamaoka N. (2014). Total purine and purine base content of common foodstuffs for facilitating nutritional therapy for gout and hyperuricemia. *Biological & Pharmaceutical Bulletin*.

[9] Beyl R. N. (2016). Update on importance of diet in gout. *The American Journal of Medicine*.

[10] Choi H. K., Atkinson K., Karlson E. W., Willett W., Curhan G. (2004). Purine-rich foods, dairy and protein intake, and the risk of gout in men. *The New England Journal of Medicine*.

[11] Day R. O., Kamel B., Kannangara D. R. W., Williams K. M., Graham G. G. (2016). Xanthine oxidoreductase and its inhibitors: relevance for gout. *Clinical Science (London, England)*.

[12] Doehner W., Landmesser U. (2011). Xanthine oxidase and uric acid in cardiovascular disease: clinical impact and therapeutic options. *Seminars in Nephrology*.

[13] Johnson R. J., Nakagawa T., Sanchez-Lozada L. G. (2013). Sugar, uric acid, and the etiology of diabetes and obesity. *Diabetes*.

[14] Kanbay M., Jensen T., Solak Y. (2016). Uric acid in metabolic syndrome: from an innocent bystander to a central player. *European Journal of Internal Medicine*.

[15] Borghi C., Rosei E. A., Bardin T. (2015). Serum uric acid and the risk of cardiovascular and renal disease. *Journal of Hypertension*.

[16] Bergamini C., Cicoira M., Rossi A., Vassanelli C. (2009). Oxidative stress and hyperuricaemia: pathophysiology, clinical relevance, and therapeutic implications in chronic heart failure. *European Journal of Heart Failure*.

[17] Zhou Y., Zhao M., Pu Z., Xu G., Li X. (2018). Relationship between oxidative stress and inflammation in hyperuricemia: analysis based on asymptomatic young patients with primary hyperuricemia. *Medicine*.

[18] Pascart T., Richette P. (2018). Investigational drugs for hyperuricemia, an update on recent developments. *Expert Opinion on Investigational Drugs*.

[19] Huang Y., Meng J., Sun B. (2017). Acupuncture for serum uric acid in patients with asymptomatic hyperuricemia: a randomized, double-blind, placebo-controlled trial. *International Journal of Cardiology*.

[20] Desideri G., Castaldo G., Lombardi A. (2014). Is it time to revise the normal range of serum uric acid levels?. *European Review for Medical and Pharmacological Sciences*.

[21] van den Berghe G., Bronfman M., Vanneste R., Hers H. G. (1977). The mechanism of adenosine triphosphate depletion in the liver after a load of fructose. A kinetic study of liver adenylate deaminase. *The Biochemical Journal*.

[22] de Oliveira E. P., Burini R. C. (2012). High plasma uric acid concentration: causes and consequences. *Diabetology and Metabolic Syndrome*.

[23] Emmerson B. T. (1974). Effect of oral fructose on urate production. *Annals of the Rheumatic Diseases*.

[24] Perez-Ruiz F., Calabozo M., Erauskin G. G., Ruibal A., Herrero-Beites A. M. (2002). Renal underexcretion of uric acid is present in patients with apparent high urinary uric acid output. *Arthritis and Rheumatism*.

[25] Pan J., Shi M., Li L. (2019). Pterostilbene, a bioactive component of blueberries, alleviates renal fibrosis in a severe mouse model of hyperuricemic nephropathy. *Biomedicine & Pharmacotherapy*.

[26] Richette P., Bardin T. (2010). Gout. *The Lancet*.

[27] Paul B. J., Anoopkumar K., Krishnan V. (2017). Asymptomatic hyperuricemia: is it time to intervene?. *Clinical Rheumatology*.

[28] Estevez-Garcia I. O., Gallegos-Nava S., Vera-Pérez E. (2018). Levels of cytokines and microRNAs in individuals with asymptomatic hyperuricemia and ultrasonographic findings of gout: a bench-to-bedside approach. *Arthritis Care & Research*.

[29] Cronstein B. N., Sunkureddi P. (2013). Mechanistic aspects of inflammation and clinical management of inflammation in acute gouty arthritis. *Journal of Clinical Rheumatology*.

[30] Bardin T., Richette P. (2017). Impact of comorbidities on gout and hyperuricaemia: an update on prevalence and treatment options. *BMC Medicine*.

[31] Chales G. (2018). How should we manage asymptomatic hyperuricemia?. *Joint Bone Spine*.

[32] Eleftheriadis T., Golphinopoulos S., Pissas G., Stefanidis I. (2017). Asymptomatic hyperuricemia and chronic kidney disease: narrative review of a treatment controversial. *Journal of Advanced Research*.

[33] Bellomo G., Selvi A. (2018). Uric acid: the lower the better?. *Contributions to Nephrology*.

[34] Bordier L., Blanchard A., Sarret D., Hérody M., Nédélec G., Duvic C. (2004). Hypouricemia, an old subject and new concepts. *Presse Médicale*.

[35] Beck L. H. (1979). Hypouricemia in the syndrome of inappropriate secretion of antidiuretic hormone. *New England Journal of Medicine*.

[36] Steele T. H. (1979). Hypouricemia. *The New England Journal of Medicine*.

[37] de Becker B., Coremans C., Chaumont M. (2019). Severe hypouricemia impairs endothelium-dependent vasodilatation and reduces blood pressure in healthy young men: a randomized, placebo-controlled, and crossover study. *Journal of the American Heart Association*.

[38] Sebesta I. (2012). Genetic disorders resulting in hyper- or hypouricemia. *Advances in Chronic Kidney Disease*.

[39] Windpessl M., Ritelli M., Wallner M., Colombi M. (2016). A novel homozygous SLC2A9 mutation associated with renal-induced hypouricemia. *American Journal of Nephrology*.

[40] Ruiz A., Gautschi I., Schild L., Bonny O. (2018). Human mutations in SLC2A9 (Glut9) affect transport capacity for urate. *Frontiers in Physiology*.

[41] Dissanayake L. V., Spires D. R., Palygin O., Staruschenko A. (2020). Effects of uric acid dysregulation on the kidney. *American Journal of Physiology. Renal Physiology*.

[42] Paller M. S., Hoidal J. R., Ferris T. F. (1984). Oxygen free radicals in ischemic acute renal failure in the rat. *The Journal of Clinical Investigation*.

[43] Ohtsubo T., Matsumura K., Sakagami K. (2009). Xanthine oxidoreductase depletion induces renal interstitial fibrosis through aberrant lipid and purine accumulation in renal tubules. *Hypertension*.

[44] Glantzounis G. K., Tsimoyiannis E., Kappas A., Galaris D. (2005). Uric acid and oxidative stress. *Current Pharmaceutical Design*.

[45] Waring W. S. (2002). Uric acid: an important antioxidant in acute ischaemic stroke. *QJM*.

[46] Ames B. N., Cathcart R., Schwiers E., Hochstein P. (1981). Uric acid provides an antioxidant defense in humans against oxidant- and radical-caused aging and cancer: a hypothesis. *Proceedings of the National Academy of Sciences of the United States of America*.

[47] Skinner K. A., White C. R., Patel R. (1998). Nitrosation of uric acid by peroxynitrite:. *The Journal of Biological Chemistry*.

[48] Ghio A. J., Kennedy T. P., Stonehuerner J. (2002). Iron regulates xanthine oxidase activity in the lung. *American Journal of Physiology. Lung Cellular and Molecular Physiology*.

[49] Tana C., Ticinesi A., Prati B., Nouvenne A., Meschi T. (2018). Uric acid and cognitive function in older individuals. *Nutrients*.

[50] Muraoka S., Miura T. (2003). Inhibition by uric acid of free radicals that damage biological molecules. *Pharmacology & Toxicology*.

[51] Simão A. N. C., Lozovoy M. A., Dichi I. (2012). The uric acid metabolism pathway as a therapeutic target in hyperuricemia related to metabolic syndrome. *Expert Opinion on Therapeutic Targets*.

[52] Yu M. A., Sánchez-Lozada L. G., Johnson R. J., Kang D. H. (2010). Oxidative stress with an activation of the renin-angiotensin system in human vascular endothelial cells as a novel mechanism of uric acid-induced endothelial dysfunction. *Journal of Hypertension*.

[53] Sautin Y. Y., Johnson R. J. (2008). Uric acid: the oxidant-antioxidant paradox. *Nucleosides, Nucleotides & Nucleic Acids*.

[54] Patterson R. A., Horsley E. T., Leake D. S. (2003). Prooxidant and antioxidant properties of human serum ultrafiltrates toward LDL: important role of uric acid. *Journal of Lipid Research*.

[55] Bagnati M., Perugini C., Cau C., Bordone R., Albano E., Bellomo G. (1999). When and why a water-soluble antioxidant becomes pro-oxidant during copper-induced low-density lipoprotein oxidation: a study using uric acid. *The Biochemical Journal*.

[56] Kadowaki D., Sakaguchi S., Miyamoto Y. (2015). Direct radical scavenging activity of benzbromarone provides beneficial antioxidant properties for hyperuricemia treatment. *Biological & Pharmaceutical Bulletin*.

[57] Cillero-Pastor B., Martin M. A., Arenas J., López-Armada M. J., Blanco F. J. (2011). Effect of nitric oxide on mitochondrial activity of human synovial cells. *BMC Musculoskeletal Disorders*.

[58] Hussain S. P., Hofseth L. J., Harris C. C. (2003). Radical causes of cancer. *Nature Reviews. Cancer*.

[59] Zagkle E., Grosiak M., Bauchinger U., Sadowska E. T. (2020). Rest-phase hypothermia reveals a link between aging and oxidative stress: a novel hypothesis. *Frontiers in Physiology*.

[60] Seddon M., Looi Y. H., Shah A. M. (2007). Oxidative stress and redox signalling in cardiac hypertrophy and heart failure. *Heart*.

[61] Sánchez-Lozada L. G., Soto V., Tapia E. (2008). Role of oxidative stress in the renal abnormalities induced by experimental hyperuricemia. *American Journal of Physiology. Renal Physiology*.

[62] Albu A., Para I., Porojan M. (2020). Uric acid and arterial stiffness. *Therapeutics and Clinical Risk Management*.

[63] McNally J. S., Davis M. E., Giddens D. P. (2003). Role of xanthine oxidoreductase and NAD(P)H oxidase in endothelial superoxide production in response to oscillatory shear stress. *American Journal of Physiology. Heart and Circulatory Physiology*.

[64] Eleftheriadis T., Pissas G., Antoniadi G., Liakopoulos V., Stefanidis I. (2018). Allopurinol protects human glomerular endothelial cells from high glucose-induced reactive oxygen species generation, p53 overexpression and endothelial dysfunction. *International Urology and Nephrology*.

[65] Rosenthal T. R., Park S. K., Kairamkonda S., Khatoon S., Pop L. M., Bobulescu I. A. (2020). Renal lipid accumulation, oxidative stress and uric acid handling in a rodent model of obesity and metabolic syndrome. *Journal of Investigative Medicine*.

[66] Corry D. B., Eslami P., Yamamoto K., Nyby M. D., Makino H., Tuck M. L. (2008). Uric acid stimulates vascular smooth muscle cell proliferation and oxidative stress via the vascular renin-angiotensin system. *Journal of Hypertension*.

[67] Sautin Y. Y., Nakagawa T., Zharikov S., Johnson R. J. (2007). Adverse effects of the classic antioxidant uric acid in adipocytes: NADPH oxidase-mediated oxidative/nitrosative stress. *American Journal of Physiology. Cell Physiology*.

[68] Ramana K. V. (2011). ALDOSE REDUCTASE: new insights for an old enzyme. *Biomolecular Concepts*.

[69] Zhang Y., Hong Q., Huang Z. (2014). ALDR enhanced endothelial injury in hyperuricemia screened using SILAC. *Cellular Physiology and Biochemistry*.

[70] Huang Z., Hong Q., Zhang X. (2017). Aldose reductase mediates endothelial cell dysfunction induced by high uric acid concentrations. *Cell Communication and Signaling: CCS*.

[71] Su H. Y., Yang C., Liang D., Liu H. F. (2020). Research advances in the mechanisms of hyperuricemia-induced renal injury. *BioMed Research International*.

[72] Cristóbal-García M., García-Arroyo F. E., Tapia E. (2015). Renal oxidative stress induced by long-term hyperuricemia alters mitochondrial function and maintains systemic hypertension. *Oxidative Medicine and Cellular Longevity*.

[73] Sanchez-Lozada L. G., Lanaspa M. A., Cristóbal-García M. (2012). Uric acid-induced endothelial dysfunction is associated with mitochondrial alterations and decreased intracellular ATP concentrations. *Nephron. Experimental Nephrology*.

[74] Arai H., Hori S., Aramori I., Ohkubo H., Nakanishi S. (1990). Cloning and expression of a cDNA encoding an endothelin receptor. *Nature*.

[75] Pacher P., Beckman J. S., Liaudet L. (2007). Nitric oxide and peroxynitrite in health and disease. *Physiological Reviews*.

[76] Davies K. J., Sevanian A., Muakkassah-Kelly S. F., Hochstein P. (1986). Uric acid-iron ion complexes. A new aspect of the antioxidant functions of uric acid. *The Biochemical Journal*.

[77] Hong Q., Wang L., Huang Z. (2020). High concentrations of uric acid and angiotensin II act additively to produce endothelial injury. *Mediators of Inflammation*.

[78] Choi Y. J., Yoon Y., Lee K. Y. (2014). Uric acid induces endothelial dysfunction by vascular insulin resistance associated with the impairment of nitric oxide synthesis. *The FASEB Journal*.

[79] Jin M., Yang F., Yang I. (2012). Uric acid, hyperuricemia and vascular diseases. *Frontiers in Bioscience*.

[80] Jing P., Shi M., Ma L., Fu P. (2020). Mechanistic insights of soluble uric acid-related kidney disease. *Current Medicinal Chemistry*.

[81] Khosla U. M., Zharikov S., Finch J. L. (2005). Hyperuricemia induces endothelial dysfunction. *Kidney International*.

[82] Zamudio-Cuevas Y., Hernández-Díaz C., Pineda C. (2015). Molecular basis of oxidative stress in gouty arthropathy. *Clinical Rheumatology*.

[83] Gliozzi M., Malara N., Muscoli S., Mollace V. (2016). The treatment of hyperuricemia. *International Journal of Cardiology*.

[84] Wilcox C. S. (2005). Oxidative stress and nitric oxide deficiency in the kidney: a critical link to hypertension?. *American Journal of Physiology. Regulatory, Integrative and Comparative Physiology*.

[85] Ndrepepa G. (2018). Uric acid and cardiovascular disease. *Clinica Chimica Acta*.

[86] Long C. L., Qin X. C., Pan Z. Y. (2008). Activation of ATP-sensitive potassium channels protects vascular endothelial cells from hypertension and renal injury induced by hyperuricemia. *Journal of Hypertension*.

[87] Mehmood A., Zhao L., Ishaq M. (2020). Anti-hyperuricemic potential of stevia (Stevia rebaudiana Bertoni) residue extract in hyperuricemic mice. *Food & Function*.

[88] Xie H., Sun J., Chen Y., Zong M., Li S., Wang Y. (2015). EGCG attenuates uric acid-induced inflammatory and oxidative stress responses by medicating the NOTCH pathway. *Oxidative Medicine and Cellular Longevity*.

[89] Battelli M. G., Bortolotti M., Polito L., Bolognesi A. (2018). The role of xanthine oxidoreductase and uric acid in metabolic syndrome. *Biochimica et Biophysica Acta - Molecular Basis of Disease*.

[90] Battelli M. G., Polito L., Bortolotti M., Bolognesi A. (2016). Xanthine oxidoreductase-derived reactive species: physiological and pathological effects. *Oxidative Medicine and Cellular Longevity*.

[91] Battelli M. G., Polito L., Bortolotti M., Bolognesi A. (2016). Xanthine oxidoreductase in cancer: more than a differentiation marker. *Cancer Medicine*.

[92] Nishino T., Okamoto K., Kawaguchi Y. (2015). The C-terminal peptide plays a role in the formation of an intermediate form during the transition between xanthine dehydrogenase and xanthine oxidase. *The FEBS Journal*.

[93] Battelli M. G., Bolognesi A., Polito L. (2014). Pathophysiology of circulating xanthine oxidoreductase: new emerging roles for a multi-tasking enzyme. *Biochimica et Biophysica Acta*.

[94] Hille R., Nishino T. (1995). Flavoprotein structure and mechanism. 4. Xanthine oxidase and xanthine dehydrogenase. *The FASEB Journal*.

[95] Kelley E. E. (2015). Dispelling dogma and misconceptions regarding the most pharmacologically targetable source of reactive species in inflammatory disease, xanthine oxidoreductase. *Archives of Toxicology*.

[96] Chung H. Y., Baek B. S., Song S. H. (1997). Xanthine dehydrogenase/xanthine oxidase and oxidative stress. *Age (Omaha)*.

[97] Stockert A. L., Shinde S. S., Anderson R. F., Hille R. (2002). The reaction mechanism of xanthine oxidase: evidence for two-electron chemistry rather than sequential one-electron steps. *Journal of the American Chemical Society*.

[98] Harrison R. (2002). Structure and function of xanthine oxidoreductase: where are we now?. *Free Radical Biology & Medicine*.

[99] Harmon D. B., Mandler W. K., Sipula I. J. (2019). Hepatocyte-specific ablation or Whole-Body inhibition of xanthine oxidoreductase in mice corrects obesity-induced systemic hyperuricemia without improving metabolic abnormalities. *Diabetes*.

[100] Battelli M. G., Polito L., Bolognesi A. (2014). Xanthine oxidoreductase in atherosclerosis pathogenesis: not only oxidative stress. *Atherosclerosis*.

[101] Battelli M. G., Bortolotti M., Polito L., Bolognesi A. (2019). Metabolic syndrome and cancer risk: the role of xanthine oxidoreductase. *Redox Biology*.

[102] Cannon P. J., Stason W. B., Demartini F. E., Sommers S. C., Laragh J. H. (1966). Hyperuricemia in primary and renal hypertension. *The New England Journal of Medicine*.

[103] Day R. O., Kannangara D. R. W., Stocker S. L., Carland J. E., Williams K. M., Graham G. G. (2017). Allopurinol: insights from studies of dose-response relationships. *Expert Opinion on Drug Metabolism & Toxicology*.

[104] Torres R. J., Puig J. G. (2007). Hypoxanthine-guanine phosophoribosyltransferase (HPRT) deficiency: Lesch-Nyhan syndrome. *Orphanet Journal of Rare Diseases*.

[105] Chen C., Lü J.-M., Yao Q. (2016). Hyperuricemia-related diseases and xanthine oxidoreductase (XOR) inhibitors: an overview. *Medical Science Monitor*.

[106] Day R. O., Graham G. G., Hicks M., McLachlan A. J., Stocker S. L., Williams K. M. (2007). Clinical pharmacokinetics and pharmacodynamics of allopurinol and oxypurinol. *Clinical Pharmacokinetics*.

[107] Kumar R., Joshi G., Kler H., Kalra S., Kaur M., Arya R. (2018). Toward an understanding of structural insights of xanthine and aldehyde oxidases: an overview of their inhibitors and role in various diseases. *Medicinal Research Reviews*.

[108] Terao M., Romão M. J., Leimkühler S. (2016). Structure and function of mammalian aldehyde oxidases. *Archives of Toxicology*.

[109] Stamp L. K., Day R. O., Yun J. (2016). Allopurinol hypersensitivity: investigating the cause and minimizing the risk. *Nature Reviews Rheumatology*.

[110] Stamp L. K., Chapman P. T. (2020). Allopurinol hypersensitivity: pathogenesis and prevention. *Best Practice & Research. Clinical Rheumatology*.

[111] Simsek M., Opperman R. C. M., Mulder C. J. J., Lambalk C. B., de Boer N. K. H. (2018). The teratogenicity of allopurinol: a comprehensive review of animal and human studies. *Reproductive Toxicology*.

[112] Stamp L. K., Chapman P. T., Palmer S. C. (2016). Allopurinol and kidney function: an update. *Joint, Bone, Spine*.

[113] Pagidipati N. J., Clare R. M., Keenan R. T., Chiswell K., Roe M. T., Hess C. N. (2018). Association of gout with long-term cardiovascular outcomes among patients with obstructive coronary artery disease. *Journal of the American Heart Association*.

[114] Vargas-Santos A. B., Peloquin C. E., Zhang Y., Neogi T. (2018). Association of chronic kidney disease with allopurinol use in gout treatment. *JAMA Internal Medicine*.

[115] Shih H. J., Kao M. C., Tsai P. S., Fan Y. C., Huang C. J. (2017). Long-term allopurinol use decreases the risk of prostate cancer in patients with gout: a population-based study. *Prostate Cancer and Prostatic Diseases*.

[116] Akhondzadeh S., Milajerdi M. R., Amini H., Tehrani-Doost M. (2006). Allopurinol as an adjunct to lithium and haloperidol for treatment of patients with acute mania: a double-blind, randomized, placebo-controlled trial. *Bipolar Disorders*.

[117] Schlesinger N., Brunetti L. (2020). Beyond urate lowering: Analgesic and anti-inflammatory properties of allopurinol. *Seminars in Arthritis and Rheumatism*.

[118] Luo J., Yan D., Li S. (2020). Allopurinol reduces oxidative stress and activates Nrf2/p62 to attenuate diabetic cardiomyopathy in rats. *Journal of Cellular and Molecular Medicine*.

[119] Grabowski B. A., Khosravan R., Vernillet L., Mulford D. J. (2011). Metabolism and excretion of [14C] febuxostat, a novel nonpurine selective inhibitor of xanthine oxidase, in healthy male subjects. *Journal of Clinical Pharmacology*.

[120] Okamoto K., Eger B. T., Nishino T., Kondo S., Pai E. F., Nishino T. (2003). An extremely potent inhibitor of xanthine oxidoreductase. Crystal structure of the enzyme-inhibitor complex and mechanism of inhibition. *The Journal of Biological Chemistry*.

[121] Becker M. A., Schumacher H. R., MacDonald P. A., Lloyd E., Lademacher C. (2009). Clinical efficacy and safety of successful longterm urate lowering with febuxostat or allopurinol in subjects with gout. *The Journal of Rheumatology*.

[122] Dalbeth N., Saag K. G., Palmer W. E. (2017). Effects of febuxostat in early Gout, Placebo-Controlled Study. *Arthritis & Rhematology*.

[123] Fukui T., Maruyama M., Yamauchi K., Yoshitaka S., Yasuda T., Abe Y. (2015). Effects of febuxostat on oxidative stress. *Clinical Therapeutics*.

[124] Becker M. A., Kisicki J., Khosravan R. (2004). Febuxostat (TMX-67), a novel, non-purine, selective inhibitor of xanthine oxidase, is safe and decreases serum urate in healthy volunteers. *Nucleosides, Nucleotides & Nucleic Acids*.

[125] Schumacher H. R. (2005). Febuxostat: a non-purine, selective inhibitor of xanthine oxidase for the management of hyperuricaemia in patients with gout. *Expert Opinion on Investigational Drugs*.

[126] Shiramoto M., Liu S., Shen Z. (2018). Verinurad combined with febuxostat in Japanese adults with gout or asymptomatic hyperuricaemia: a phase 2a, open-label study. *Rheumatology (Oxford)*.

[127] Davies K., Bukhari M. A. S. (2018). Recent pharmacological advances in the management of gout. *Rheumatology (Oxford)*.

[128] Suresh E., Das P. (2012). Recent advances in management of gout. *QJM*.

[129] Kimura K., Hosoya T., Uchida S. (2018). Febuxostat therapy for patients with stage 3 CKD and asymptomatic hyperuricemia: a randomized trial. *American Journal of Kidney Diseases*.

[130] Li S. Y., Zhang T. J., Wu Q. X., Olounfeh K. M., Zhang Y., Meng F. H. (2020). Synthesis and biological evaluation of 5-benzyl-3-pyridyl-1H-1,2,4-triazole derivatives as xanthine oxidase inhibitors. *Medicinal Chemistry*.

[131] Hosoya T., Sasaki T., Ohashi T. (2017). Clinical efficacy and safety of topiroxostat in Japanese hyperuricemic patients with or without gout: a randomized, double-blinded, controlled phase 2b study. *Clinical Rheumatology*.

[132] Ojha R., Singh J., Ojha A., Singh H., Sharma S., Nepali K. (2017). An updated patent review: xanthine oxidase inhibitors for the treatment of hyperuricemia and gout (2011-2015). *Expert Opinion on Therapeutic Patents*.

[133] Hosoya T., Sasaki T., Hashimoto H., Sakamoto R., Ohashi T. (2016). Clinical efficacy and safety of topiroxostat in Japanese male hyperuricemic patients with or without gout: an exploratory, phase 2a, multicentre, randomized, double-blind, placebo-controlled study. *Journal of Clinical Pharmacy and Therapeutics*.

[134] Hosoya T., Ohno I., Nomura S. (2014). Effects of topiroxostat on the serum urate levels and urinary albumin excretion in hyperuricemic stage 3 chronic kidney disease patients with or without gout. *Clinical and Experimental Nephrology*.

[135] Katsuyama H., Yanai H., Hakoshima M. (2019). Renoprotective effect of xanthine oxidase inhibitor, topiroxostat, Topiroxostat. *Journal of Clinical Medical Research*.

[136] Kawamorita Y., Shiraishi T., Tamura Y. (2017). Renoprotective effect of topiroxostat via antioxidant activity in puromycin aminonucleoside nephrosis rats. *Physiological Reports*.

[137] Sezai A., Unosawa S., Taoka M., Osaka S., Sekino H., Tanaka M. (2020). Changeover trial of febuxostat and topiroxostat for hyperuricemia with cardiovascular disease: sub-analysis for chronic kidney disease (TROFEO CKD trial). *Annals of Thoracic and Cardiovascular Surgery*.

[138] Luna G., Dolzhenko A. V., Mancera R. L. (2019). Inhibitors of xanthine oxidase: scaffold diversity and structure-based drug design. *ChemMedChem*.

[139] Sousa T., Morato M., Fernandes E., Carvalho F., Albino-Teixeira A. (2004). Xanthine oxidase inhibition by 1,3-dipropyl-8-sulfophenylxanthine (DPSPX), an antagonist of adenosine receptors. *Journal of Enzyme Inhibition and Medicinal Chemistry*.

[140] Biagi G., Giorgi I., Pacchini F., Livi O., Scartoni V. (2001). 2-Alkyloxyalkylthiohypoxanthines as new potent inhibitors of xanthine oxidase. *Farmaco*.

[141] Baker B., Wood W. F. (1968). Irreversible enzyme inhibitors. CXXIII. Candidate irreversible inhibitors of guanine deaminase and xanthine oxidase derived from 9-phenylguanine substituted with a terminal sulfonyl fluoride. *Journal of Medicinal Chemistry*.

[142] Oettl K., Reibnegger G. (1999). Pteridines as inhibitors of xanthine oxidase: structural requirements. *Biochimica et Biophysica Acta*.

[143] Nepali K., Agarwal A., Sapra S. (2011). N_ -(1,3-Diaryl-3-oxopropyl)amides as a new template for xanthine oxidase inhibitors. *Bioorganic & Medicinal Chemistry*.

[144] Kumar D., Kaur G., Negi A., Kumar S., Singh S., Kumar R. (2014). Synthesis and xanthine oxidase inhibitory activity of 5,6-dihydropyrazolo/pyrazolo[1,5- _c_ ]quinazoline derivatives. *Bioorganic Chemistry*.

[145] Rodrigues M. V., Barbosa A. F., da Silva J. F. (2016). 9-Benzoyl 9-deazaguanines as potent xanthine oxidase inhibitors. *Bioorganic & Medicinal Chemistry*.

[146] Nile S. H., Kumar B., Park S. W. (2013). In vitro evaluation of selected benzimidazole derivatives as an antioxidant and xanthine oxidase inhibitors. *Chemical Biology & Drug Design*.

[147] Sharma S., Sharma K., Ojha R. (2014). Microwave assisted synthesis of naphthopyrans catalysed by silica supported fluoroboric acid as a new class of non purine xanthine oxidase inhibitors. *Bioorganic & Medicinal Chemistry Letters*.

[148] Chen S., Zhang T., Wang J. (2015). Synthesis and evaluation of 1-hydroxy/methoxy-4-methyl-2-phenyl-1 _H_ -imidazole-5-carboxylic acid derivatives as non-purine xanthine oxidase inhibitors. *European Journal of Medicinal Chemistry*.

[149] Song J. U., Jang J. W., Kim T. H. (2016). Structure-based design and biological evaluation of novel 2-(indol-2-yl) thiazole derivatives as xanthine oxidase inhibitors. *Bioorganic & Medicinal Chemistry Letters*.

[150] Ishibuchi S., Morimoto H., Oe T. (2001). Synthesis and structure-activity relationships of 1-phenylpyrazoles as xanthine oxidase inhibitors. *Bioorganic & Medicinal Chemistry Letters*.

[151] Hashimoto T., Fukunari A., Yamada I., Yanaka N., Chen D., Kato N. (2005). Y-700, a novel inhibitor of xanthine oxidase, suppresses the development of colon aberrant crypt foci and cell proliferation in 1,2-dimethylhydrazine-treated mice. *Bioscience, Biotechnology, and Biochemistry*.

[152] Mehmood A., Ishaq M., Zhao L. (2019). Natural compounds with xanthine oxidase inhibitory activity: a review. *Chemical Biology & Drug Design*.

[153] Qamar H., Rehman S., Chauhan D. K. (2019). Current status and future perspective for research on medicinal plants with anticancerous activity and minimum cytotoxic value. *Current Drug Targets*.

[154] Cos P., Ying L., Calomme M. (1998). Structure-activity relationship and classification of flavonoids as inhibitors of xanthine oxidase and superoxide scavengers. *Journal of Natural Products*.

[155] Shen L., Ji H. F. (2009). Insights into the inhibition of xanthine oxidase by curcumin. *Bioorganic & Medicinal Chemistry Letters*.

[156] Zhang C., Wang R., Zhang G., Gong D. (2018). Mechanistic insights into the inhibition of quercetin on xanthine oxidase. *International Journal of Biological Macromolecules*.

[157] Ding X., Ouyang M.-A., Shen Y.-S. (2015). Evaluation of anti-MRSA and xanthine oxidase inhibition activities of phenolic constituents from Plumula nelumbinis. *Journal of Chemistry*.

[158] Hatano T., Yasuhara T., Yoshihara R., Agata I., Noro T., Okuda T. (1990). Effects of interaction of tannins with co-existing substances. VII. Inhibitory effects of tannins and related polyphenols on xanthine oxidase. *Chemical & Pharmaceutical Bulletin (Tokyo)*.

[159] Lu J. M., Yao Q., Chen C. (2013). 3,4-Dihydroxy-5-nitrobenzaldehyde (DHNB) is a potent inhibitor of xanthine oxidase: a potential therapeutic agent for treatment of hyperuricemia and gout. *Biochemical Pharmacology*.

[160] Mizuno Y., Yamamotoya T., Nakatsu Y. (2019). Xanthine oxidase inhibitor febuxostat exerts an anti-inflammatory action and protects against diabetic nephropathy development in KK-Ay obese diabetic mice. *International Journal of Molecular Sciences*.

